# Nafamostat-Mediated Inhibition of SARS-CoV-2 Ribosomal Frameshifting Is Insufficient to Impair Viral Replication in Vero Cells. Comment on Munshi et al. Identifying Inhibitors of −1 Programmed Ribosomal Frameshifting in a Broad Spectrum of Coronaviruses. *Viruses* 2022, *14*, 177

**DOI:** 10.3390/v14071526

**Published:** 2022-07-13

**Authors:** Niklas Jäger, Markus Hoffmann, Stefan Pöhlmann, Nadine Krüger

**Affiliations:** 1 Infection Biology Unit, German Primate Center–Leibniz Institute for Primate Research, 37077 Göttingen, Germany; njaeger@dpz.eu (N.J.); mhoffmann@dpz.eu (M.H.); spoehlmann@dpz.eu (S.P.); 2 Faculty of Biology and Psychology, Georg-August-University, 37073 Göttingen, Germany

Severe acute respiratory syndrome coronavirus 2 (SARS-CoV-2) is the causative agent of the ongoing coronavirus disease 2019 (COVID-19) pandemic, which has been reported to have caused 18.2 million deaths globally until the end of 2021 [1]. Vaccine hesitancy and the emergence of variants that evade antibody responses induced by vaccination and infection, including the currently dominating Omicron variant [2,3,4,5], demonstrate the urgent need for efficient treatment options. At present, monoclonal antibodies, Molnupiravir and Nirmatrelvir, are available for therapy [6,7,8]. However, these agents need to be administered early after infection, and some are not suitable for certain patient groups, indicating that novel antivirals are still needed. One approach to obtaining new drugs is the repurposing of existing drugs used for the treatment of diseases other than COVID-19.

Nafamostat is a serine protease inhibitor that is used in Japan for the treatment of acute pancreatitis and disseminated intravascular coagulation [9,10]. With regard to SARS-CoV-2, it has been demonstrated that nafamostat inhibits viral entry into cells by blocking the activity of TMPRSS2 [11], a cellular protease that primes the SARS-CoV-2 spike protein [11,12,13]. A recent study published in *Viruses* [14] showed that Nafamostat also inhibited −1 programmed ribosomal frameshifting (−1PRF) of SARS-CoV-2, a process that is required for expression of the viral ORF1b (Figure 1a) protein and viral replication [15,16].

Our previous studies showed that Nafamostat inhibits SARS-CoV-2 entry by blocking TMPRSS2 and established the concept that Nafamostat should only exert anti-SARS-CoV-2 activity in cells for which viral entry depends on TMPRSS2 activity [11]. However, our studies were mainly carried out with a surrogate system that measures SARS-CoV-2 entry but not the subsequent steps in viral replication. As a consequence, we would have missed the antiviral activity of Nafamostat that was due to the blockade of −1PRF. Therefore, we sought to confirm that Nafamostat inhibits −1PRF, as suggested by the study by Munshi and colleagues [14], and to determine whether inhibition of −1PRF translates into the blockade of SARS-CoV-2 infection in cells that allow for TMPRSS2-independent entry.

We analyzed the inhibition of −1PRF using a dual-luciferase system similar to the one employed by Munshi and colleagues. In this system, the open reading frames for firefly (FLuc) and *Renilla reniformis* luciferase (RLuc) are separated by the −1PRF element of SARS-CoV-2, and FLuc expression depends on −1PRF (Figure 1a). We employed Vero 76 cells (African green monkey, kidney) for our studies since these cells allow for TMPRSS2-independent viral entry and are frequently used to grow SARS-CoV-2 [17]. As a control for inhibition of −1PRF, we transiently overexpressed the cellular factor shiftless (SFL), which blocks −1PRF of human immunodeficiency virus 1 (HIV) and SARS-CoV-2 [18,19,20,21], and a splice variant of SFL, termed SFL short (SFLS), which is unable to block −1PRF in the context of HIV infection [18]. Expression of SFL inhibited −1PRF in Vero 76 cells by roughly 50%, while expression of SFLS had no effect (Figure 1b), as expected. At the highest concentration, 100 µM, Nafamostat inhibited −1PRF by approximately 20% (Figure 1b), and this concentration was previously shown not to exert undesired cytotoxic effects [11]. Thus, the effects observed were specific and confirmed that Nafamostat is a −1PRF inhibitor.

We next analyzed whether Nafamostat inhibits SARS-CoV-2 infection of Vero 76 and Calu-3 cells. Nafamostat did not reduce infection of Vero 76 cells, even at a concentration of 100 µM (Figure 2) that inhibited −1PRF. In contrast, Nafamostat efficiently reduced infection of Calu-3 cells (Figure 2), for which viral entry depends on TMPRSS2 activity.

Our results confirm that Nafamostat can inhibit −1PRF. We note that Munshi and colleagues observed inhibition of −1PRF in the presence of 20 µM Nafamostat while we detected inhibition only in the presence of 100 µM Nafamostat. This difference might be due to the cell systems used–we employed Vero 76 cells for our study while Munshi and colleagues examined A549 cells, a human lung cell line that has been reported to be only poorly permissive to SARS-CoV-2 infection [22,23,24] and, therefore, did not allow studies focusing on viral replication efficiency. Importantly, the same Nafamostat concentration that inhibited −1PRF in Vero 76 cells did not block SARS-CoV-2 infection of these cells, although it reduced infection of a control cell line, Calu-3, by more than a thousand-fold. These results suggest that inhibition of −1PRF by Nafamostat observed in cell-free and cell-based reporter assays [14] might not translate into antiviral activity. The underlying reasons are at present unclear, and one can speculate that potential differences in RNA structures of the −1PRF element in the context of the reporter construct and the SARS-CoV-2 genome might contribute. Further, we cannot exclude that somewhat more potent inhibition of −1PRF can be detected in other cell systems and may result in low levels of antiviral activity. Importantly, Nafamostat has a short half-life time due to rapid hydrolysis by blood and liver esterases [25,26,27]. Therefore, it remains unclear whether Nafamostat concentrations suitable to block −1PRF can be attained in patients receiving Nafamostat via continuous infusion, the approved route of Nafamostat administration [28,29], or upon topical application, a recently pursued approach to COVID-19 therapy [30]. Nevertheless, although Nafamostat-mediated inhibition of −1PRF did not translate into reduced SARS-CoV-2 replication in our study, interference with SARS-CoV-2 −1PRF by more potent compounds could still represent a promising antiviral strategy.

## Figures and Tables

**Figure 1:**
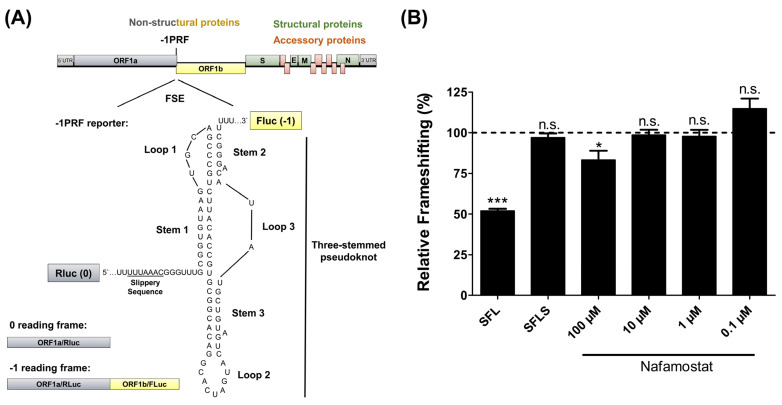
Nafamostat inhibits SARS-CoV-2 −1PRF. (**A**) Top panel: Schematic illustration of the SARS-CoV-2 genome, including the site of −1PRF and the frameshifting stimulation element (FSE). Bottom panel: For quantification of −1PRF, the FSE sequence, including the heptanucleotide slippery sequence, and the three-stemmed pseudoknot sequence (GenBank: NC_004718) were inserted between the coding sequences for *Renilla reniformis* luciferase (RLuc) (0 reading frame) and firefly luciferase (FLuc) (−1 reading frame). As a consequence, FLuc is only translated if −1PRF occurs, with the ratio between FLuc and RLuc activities indicating the efficiency of −1PRF. In order to determine the maximum signal for frameshifting, the heptanucleotide slippery sequence and the FSE were removed and FLuc was set into the same reading frame as RLuc (0 frame) (LucMax reporter). (**B**) Vero 76 cells were treated with the indicated concentrations of Nafamostat for 1 h. DMSO treatment served as a control. Subsequently, the treated cells were transfected with a plasmid encoding the SARS-CoV-2 −1PRF reporter cassette or were cotransfected with the reporter plasmid and pQCXIP-plasmids encoding SFL or SFLS under the control of a CMV promotor using Lipofectamine 2000 (Thermo Fisher Scientific, Waltham, MA, USA). To exclude unspecific effects of Nafamostat on translation (0 frame product) cells were transfected with LucMax reporter and were treated with Nafamostat as described for the −1PRF reporter. At 12 h post-transfection, the medium was replaced by fresh medium again containing Nafamostat at the indicated concentrations and cells were incubated for an additional 36 h. At 48 h post-transfection, RLuc and FLuc signals were quantified using a luminometer. The ratio of FLuc versus RLuc signals measured for the −1PRF reporter and the LucMax reporter was determined. Next, the results obtained for the −1PRF reporter were normalized against the respective results measure for the LucMax reporter. Finally, relative frameshifting measured upon treatment with DMSO was set as 100%. The average of six biological replicates carried out with technical triplicates is shown. Error bars indicate the standard error of the mean (SEM). Statistical significance was assessed by one-way analysis of variance (ANOVA) with Dunnett’s multiple comparison test (*, *p* ≤ 0.05; ***, *p* ≤ 0.001).

**Figure 2:**
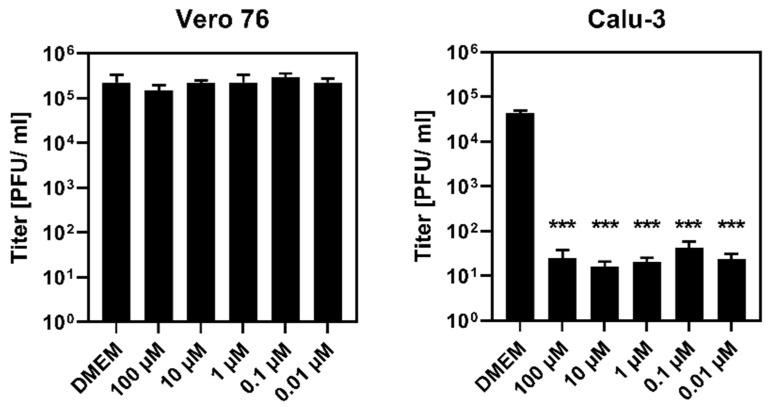
Nafamostat inhibits SARS-CoV-2 infection of Calu-3 but not Vero 76 cells. Vero 76 and Calu-3 cells were incubated with the indicated 10-fold serial dilutions of Nafamostat for 1 h prior to infection with SARS-CoV-2, Pango lineage B.1.513, at a multiplicity of infection of 0.01 for 1 h. After virus inoculation, cells were washed and further incubated with Nafamostat for 24 h. Virus-containing supernatants were harvested and viral titers were determined by titration on Vero E6 cells. Titers are shown as plaque forming units (PFU)/mL. The graphs show mean ± SEM of three independent biological replicates. Statistical significance of differences between viral titers of control-treated cells (DMEM) and cells incubated with Nafamostat was analyzed by one-way analysis of variance (ANOVA) with Dunnett’s posttest (*p* > 0.05, not significant [not indicated]; ***, *p* ≤ 0.001).
